# Investigating the Association Between Seven Sleep Traits and Nonalcoholic Fatty Liver Disease: Observational and Mendelian Randomization Study

**DOI:** 10.3389/fgene.2022.792558

**Published:** 2022-05-17

**Authors:** Hong Fan, Zhenqiu Liu, Xin Zhang, Huangbo Yuan, Xiaolan Zhao, Renjia Zhao, Tingting Shi, Sheng Wu, Yiyun Xu, Chen Suo, Xingdong Chen, Tiejun Zhang

**Affiliations:** ^1^ Department of Epidemiology, School of Public Health, Fudan University, Shanghai, China; ^2^ Key Laboratory of Public Health Safety, Ministry of Education, Fudan University, Shanghai, China; ^3^ Fudan University Taizhou Institute of Health Sciences, Taizhou, China; ^4^ State Key Laboratory of Genetic Engineering and Collaborative Innovation Center for Genetics and Development, School of Life Sciences, Fudan University, Shanghai, China; ^5^ Department of Chronic Diseases Prevention, Taizhou Center for Disease Control and Prevention, Jiangsu, China; ^6^ Human Phenome Institute, Fudan University, Shanghai, China

**Keywords:** Mendelian randomization, nonalcoholic fatty liver disease, sleep duration, insomnia, easiness of getting up in the morning

## Abstract

**Background and Aim:** Aberrant sleep parameters are associated with the risk of nonalcoholic fatty liver disease (NAFLD). However, existing information is inconsistent among studies and involves reverse causation. Therefore, we aimed to investigate the observational associations and causations between sleep traits and NAFLD.

**Methods:** We performed multivariable regression to assess observational associations of seven sleep traits (sleep duration, easiness of getting up in the morning, chronotype, nap during day, snoring, insomnia, and narcolepsy), and NAFLD in the UK Biobank (1,029 NAFLD). The Cox proportional hazards model was applied to derive hazard ratios and 95% confidence intervals (CIs). Furthermore, a bidirectional two-sample Mendelian randomization (MR) approach was used to explore the causal relationships between sleep traits and NAFLD.

**Results:** In the multivariable regression model adjusted for potential confounders, getting up in the morning not at all easy (HR, 1.51; 95% CI, 1.27–1.78) and usually insomnia (HR, 1.46; 95% CI, 1.21–1.75) were associated with the risk of NAFLD. Furthermore, the easiness of getting up in the morning and insomnia showed a dose–response association with NAFLD (P_trend_ <0.05). MR analysis found consistent causal effects of NAFLD on easiness of getting up in the morning (OR, 0.995; 95% CI, 0.990–0.999; *p* = 0.033) and insomnia (OR, 1.006; 95% CI, 1.001–1.011; *p* = 0.024). These results were robust to weak instrument bias, pleiotropy, and heterogeneity.

**Conclusions:** Findings showed consistent evidence of observational analyses and MR analyses that trouble getting up in the morning and insomnia were associated with an increased risk of NAFLD. Bidirectional MR demonstrated causal effects of NAFLD on sleep traits.

## Introduction

Nonalcoholic fatty liver disease (NAFLD) is the leading cause of chronic liver diseases worldwide (Younossi, 2017; Estes et al., 2018). The global prevalence of NAFLD is approximately 25% (Younossi et al., 2016) and is estimated to reach 56% by 2030 (Estes et al., 2018). The etiology of NAFLD involves multiple potential factors, the most common risk factors are overweight, type 2 diabetes mellitus (T2DM), high cholesterol, and metabolic syndrome (Liu et al., 2020). However, sleep-related problems have attracted less attention than other factors. Sleep-related problems involve a set of traits, including sleep duration, insomnia, nap during day, and easiness of getting up in the morning. Studies have shown that the traits are associated with various health effects (Richmond et al., 2019; Zheng et al., 2019; Cai et al., 2020; Park et al., 2020; Titova et al., 2020).

Sleep-related problems can disrupt the circadian clock and thereby the body metabolism (Sharma and Kavuru, 2010). Approximately 90% of patients with NAFLD have more than one feature of metabolic syndrome (Paschos and Paletas, 2009). Therefore, we hypothesized an association between sleep traits and NAFLD. However, there is limited information on the association between sleep traits and NAFLD. Previous study indicated that sleep disturbance was common in patients with NAFLD (Akram et al., 2021). In addition, obstructive sleep apnea, characterized by snoring and daytime naps, can influence the histological severity of NAFLD (Musso et al., 2013; Orr et al., 2020). Several prior studies have shown that both short and long sleep (Liu et al., 2016; Peng et al., 2017; Kim et al., 2018; Okamura et al., 2019), insomnia (Wijarnpreecha et al., 2017), daytime naps of more than 0.5 h (Peng et al., 2017) and snoring (Wang et al., 2020) were associated with an increased risk of NAFLD. However, most studies have focused on sleep duration (Peng et al., 2017; Kim et al., 2018; Okamura et al., 2019), while little has been reported on other sleep traits and NAFLD. Moreover, existing evidence on sleep traits and NAFLD was predominantly from cross-sectional studies with a limited number of participants and may be biased with obesity, type 2 diabetes mellitus (T2DM), and other potential confounders, while none of them implicated a clear causal effect of sleep traits on NAFLD.

The MR method using genetic variants as instrumental variables (IVs) for risk factors has been applied to examine the causal relationship between the risk factors and diseases. As the genotypes are robust and developed before exposure factors, MR is less susceptible to measurement errors, confounding, and reverse causation than conventional observational studies (Smith et al., 2007). In general, a rational MR design could provide more reliable evidence than observational studies under specific assumptions for IVs (Bowden et al., 2015; Zuccolo and Holmes, 2017), which can be used to guide clinical practice. This study conducted rigorous screening to ensure that the genetic variants met the central assumptions (Bowden et al., 2015). Furthermore, since many aspects of sleep are heritable and genes influence traits such as snoring and insomnia, genetic variants could empower the causal inference by reducing the potential confounding.

There is a growing interest in understanding whether different sleep traits could serve as etiological factors for NAFLD. Given the scarcity of information on the relationship between sleep traits and NAFLD, we investigated the association between seven sleep traits and NAFLD. We conducted an observational association analysis in the UK Biobank. To disentangle the causation between sleep traits and NAFLD, we combined external genome-wide association study (GWAS) summary data to conduct a bidirectional two-sample MR analysis.

## Methods

### Study Population

The individual-level analyses are based on data from the UK Biobank, which is a population-based prospective cohort comprising epidemiological and genetic data of 502,505 individuals aged between 37 and 73 years recruited across the United Kingdom between 2006 and 2010 (Sudlow et al., 2015). We included available information on seven self-reported sleep traits (sleep duration, chronotype, insomnia, nap during day, easiness of getting up in the morning, snoring, and narcolepsy) from 409,605 European ancestry individuals in the UK Biobank. Because sleep apnea is accompanied by snoring, trouble getting up in the morning, and interference with sleep duration, participants diagnosed with sleep apnea were excluded from this study. In addition, participants with missing covariates were excluded from the analysis. NAFLD was defined based on diagnosis codes from hospitalization records (data fields 41,270 and 41,271) following previous criteria (Bianco et al., 2020). Individuals with ICD-9 code 571.8 and ICD-10 code K76 but without hepatitis B or C infection or other specific liver diseases were characterized as NAFLD cases (Liu et al., 2020). Individuals with ICD-10 code G47.3 were characterized as apnea (Han et al., 2021).

The GWAS summary statistics of sleep traits were obtained from the UK Biobank and extracted from the MR Base platform, including sleep duration (*n* = 460,099), easiness of getting up in the morning (*n* = 461,658), nap during day (*n* = 337,074), and insomnia (*n* = 337,074). The GWAS summary statistics of NAFLD included 1,483 biopsied NAFLD cases and 17,781 controls and were downloaded from the GWAS Catalog (https://www.ebi.ac.uk/gwas/). The characteristics of the GWASs are listed in Supplementary Table S1. The study flowchart is showed in Supplementary Figure S1.

Participants enrolled in the UK Biobank signed the consent forms. This study was conducted under application number 58484.

### Sleep Traits

Sleep traits were collected using a standardized touchscreen questionnaire in the UK Biobank (Bycroft et al., 2018). Sleep duration was assessed in the question “about how many hours do you get in every 24 h? (Please include naps).” Binary variables for short sleep duration (<7 h vs. 7–8 h) and long sleep duration (>8 h vs. 7–8 h) were also derived. Easiness of getting up in the morning was assessed in the question “On an average day, how easy do you find getting up in the morning?” with one of six possible answers: “Not at all easy,” “Not very easy,” “Fairly easy,” “Very easy,” “Do not Know,” and “Prefer not to answer.” Chronotype was assessed in the question “Do you consider yourself to be?” with one of the six possible answers: “Definitely a ‘morning’ person,” “More a ‘morning’ than ‘evening’ person,” “More of an ‘evening’ than a ‘morning’ person,” “Definitely an ‘evening’ person,” “Do not know” and “Prefer not to answer.” Nap during day was assessed in the question “Do you have a nap during day?” with one of the four possible answers: “Never/rarely,” “Sometimes,” “Usually,” and “Prefer not to answer.” Insomnia was assessed in the question “Do you have trouble falling asleep at night or do you wake up in the middle of the night?” with one of four possible answers “Never/rarely,” “Sometimes,” “Usually,” and “Prefer not to answer.” Snoring was assessed in the question “Does your partner or a close relative or friend complain about your snoring?” with one of four possible answers “Yes,” “No,” “Do not know,” and “Prefer not to answer.” Narcolepsy was assessed in the question “How likely are you to doze off or fall asleep during the daytime when you don’t mean to? (e.g., when working, reading, or driving)” with one of five possible answers “Never/rarely,” “Sometimes,” “Often,” “Prefer not to answer,” and “Do not know.”

### Genetic Variants

Single nucleotide polymorphisms (SNPs) associated with insomnia and NAFLD identified in the published largest GWASs (Supplementary Tables S2, S3) were employed as IVs in the bidirectional MR analysis (Dashti et al., 2019; Jones et al., 2019; Lane et al., 2019; Anstee et al., 2020).

All of the SNPs that we employed as IVs were robustly and independently (the physical distance between each gene was greater than 10,000 KB and linkage disequilibrium (LD) *R* ^2^ < 0.01) associated with sleep traits and NAFLD (*p* < 5 × 10^–8^) and had a minor allele frequency greater than 0.01 in the GWAS. A total of 57 and 12 target SNPs were utilized as the IVs for insomnia and NAFLD, respectively. If target SNPs were not available in the outcome GWAS summary statistics, we used the proxy SNPs that were in high LD (*r* ^2^ > 0.8) with the SNPs of interest. The European samples from the 1,000 Genomes Project reference panel were adopted to estimate LD between the chosen SNPs. We then harmonized the direction of the effects between exposure and outcome associations.

### Statistical Analysis

#### Observational Analysis

Potential confounders were identified by reviewing published articles (Kasturiratne et al., 2013; Mansour et al., 2019; Orr et al., 2020). Alanine aminotransferase (ALT), glutamic transaminase (AST), waist circumference, hip circumference, body mass index (BMI), T2DM, hypertension, high-density lipoprotein (HDL), low-density lipoprotein (LDL), triglyceride (TG), total cholesterol (TC), glucose, and smoking status were included. Continuous variables are presented as mean ± standard deviation, and categorical variables are reported as numbers and percentages. Chi-square test, Student’s t-test, and Fisher’s exact test were employed to estimate the differences as appropriate. A generalized additive model was applied to plot the association between sleep duration (<3 and >15 h was excluded) and the NAFLD odds.

A separate multivariable Cox proportional hazards model was used to estimate the association between the seven sleep traits and NAFLD and test the trend separately. In the Cox proportional hazards model, covariates were set in three ways: model 1 was adjusted for age and sex, and model 2 was adjusted for age, sex, BMI, T2DM, and hypertension. Model 3 was fully adjusted for 15 potential confounders identified in the literature. Considering the multiple testing, we used the false-positive rate (FDR) method to adjust the *p-*value in the multivariable regression analyses, and FDR-adjusted *p*-value under 0.05 was considered statistically significant in observational analyses.

#### Mendelian Randomization Analysis

We conducted a bidirectional two-sample MR analysis to assess whether sleep traits have causal effects on NAFLD, and conversely, if NAFLD has a causal effect on sleep traits. There are various methods to estimate the effect in a two-sample MR analysis, each of which has a different requirement for IVs (Bowden et al., 2015; Bowden et al., 2016a; Bowden et al., 2016b). The inverse variance weighted (IVW) method with multiplicative random effects is an efficient analysis method with valid IVs, and it accounts for heterogeneity in the variant-specific causal estimates (Lin et al., 2021). In this case, we adopted the IVW method as the main method to estimate the effect between sleep traits and NAFLD, and adopted the MR-Egger, weighted median, simple mode, and weighted mode methods as sensitivity analyses. We tested the MR assumption that the variants were strongly associated with sleep traits and NAFLD by calculating the mean *F* statistic across all IVs for insomnia and NAFLD. An *F* statistic greater than 10 is an acceptable threshold for considering a genetic variant to be strong enough for MR analyses (Burgess and Thompson, 2011; Bull et al., 2020).

#### Sensitivity Analysis

Cochran’s Q test was applied to estimate the heterogeneity of individual genetic variants in both the MR-Egger (Rees et al., 2017) and IVW (Burgess and Thompson, 2015) methods. Heterogeneity was considered absent if the *p-*value was greater than 0.05.

The MR-Egger intercept test was used to estimate the directional pleiotropy. In addition, we considered the absence of pleiotropic effects if the intercept was not significantly different from zero (*p* > 0.05) (Zhang et al., 2020). We also applied the MR-Pleiotropy Residual Sum and Outlier (MR-PRESSO) global test (Verbanck et al., 2018) to evaluate horizontal pleiotropy and identify outlier variants.

The leave-one-out analysis by MR-Egger and IVW was performed to test whether the overall results were driven from an individual SNP by removing single SNPs. In addition, the IVW and MR-Egger methods were used to perform a sensitivity analysis of SNPs. Therefore, the MR-Egger estimate is unbiased, provided that the genetic instrument is not dependent on pleiotropic effects.

All statistical analyses were performed using R version 3.6.0 (R Development Core Team, Vienna, Austria). We mainly used the Mendelian Randomization (Yavorska and Burgess, 2017), Two Sample MR (Hemani et al., 2018), and the MR-PRESSO (Verbanck et al., 2018) packages.

## Results

### Baseline Characteristics of Participants

A total of 1,029 NAFLD cases and 272,063 healthy participants were included in the final analyses. The baseline characteristics of the study population are shown in Table1. Briefly, the NAFLD cases were older (mean age, 57.97 vs. 56.80 years) and had a higher mean BMI (31.22 vs. 27.32) than the non-NAFLD cases. In addition, the NAFLD cases showed a higher value of ALT, AST, waist circumference, hip circumference, BMI, HDL, LDL, TG, TC, glucose, and HbA1c than the healthy controls and presented a higher prevalence of current smoking, T2DM, and primary hypertension than non-NAFLD individuals.

**TABLE 1:** Baseline characteristics of the study population.

Variable	NAFLD (1,029)	Non-NAFLD (271,632)	*χ* ^*2*^/t	*p*
Age (years, mean ± SD)	57.97 ± 7.50	56.80 ± 8.02	5.03	<0.001
Percentage of male (%)	46.71%	45.31%	0.76	0.384
BMI (kg/m^2^, mean ± SD)	31.22 ± 5.19	27.32 ± 4.63	24.18	<0.001
Waist circumference (cm, mean ± SD)	100.10 ± 12.28	89.96 ± 13.22	26.48	<0.001
Hip circumference (cm, mean ± SD)	108.85 ± 10.81	103.28 ± 8.94	16.54	<0.001
ALT (IU/L, mean ± SD)	36.30 ± 23.57	23.41 ± 14.03	17.57	<0.001
AST (IU/L, mean ± SD)	32.84 ± 16.86	26.10 ± 10.09	12.84	<0.001
Glucose (mmol/L, mean ± SD)	5.59 ± 1.86	5.10 ± 1.16	8.46	<0.001
HDL (mmol/L, mean ± SD)	1.27 ± 0.33	1.46 ± 0.38	18.29	<0.001
LDL (mmol/L, mean ± SD)	3.48 ± 0.91	3.58 ± 0.87	3.61	<0.001
TG (mmol/L, mean ± SD)	2.31 ± 1.25	1.74 ± 1.00	14.54	<0.001
TC (mmol/L, mean ± SD)	5.52 ± 1.21	5.73 ± 1.14	5.46	<0.001
HbA1c (mmol/mol, mean ± SD)	39.51 ± 10.62	35.81 ± 6.24	11.18	<0.001
T2DM* (%)	282 (27.27%)	258829 (4.98%)	1861.60	<0.001
Primary hypertension* (%)	590 (57.06%)	58307 (21.41%)	772.65	<0.001
Smoking status				
Never	449 (43.63%)	148088 (54.52%)		
Previous	430 (41.79)	96450 (35.51%)	55.40	<0.001
Current	150 (14.58%)	27094 (9.97%)		

*T2DM is defined by ICD-10 diagnosis codes E11.

*Primary hypertension is defined by ICD-10 diagnosis codes I10.

BMI, body mass index; T2DM, type 2 diabetes mellitus; HDL, high-density lipoprotein; LDL, low-density lipoprotein; TG, triglyceride; TC, total cholesterol; ALT, alanine aminotransferase; AST, aspartate aminotransferase.

### Observational Analysis

**FIGURE 1:**
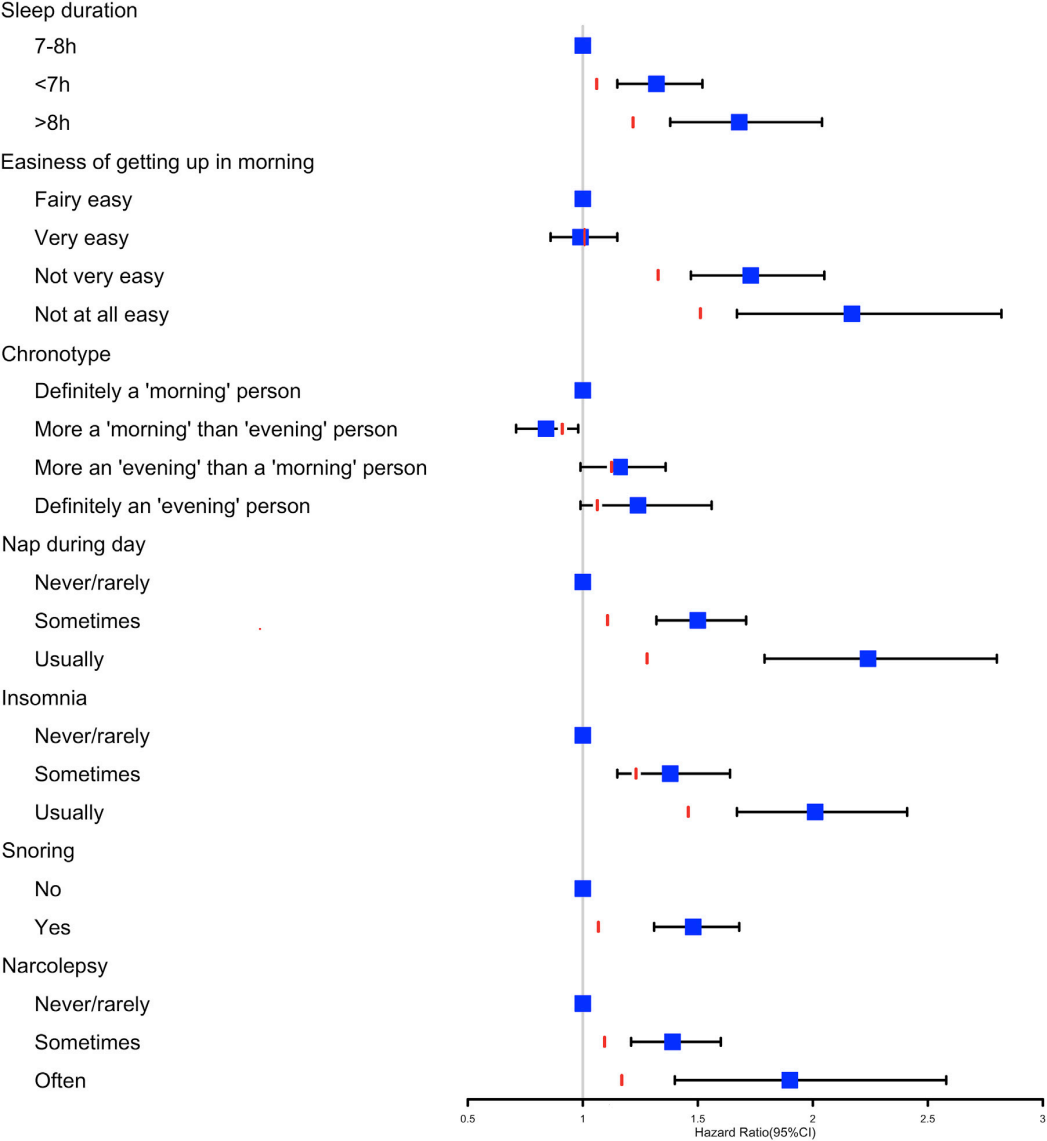
Cox proportional hazards model adjusted by age and sex analysis used to determine the association between the seven sleep traits and nonalcoholic fatty liver disease. The red line represents the HR estimates of the fully adjusted model 3 for each sleep variable.

**FIGURE 2:**
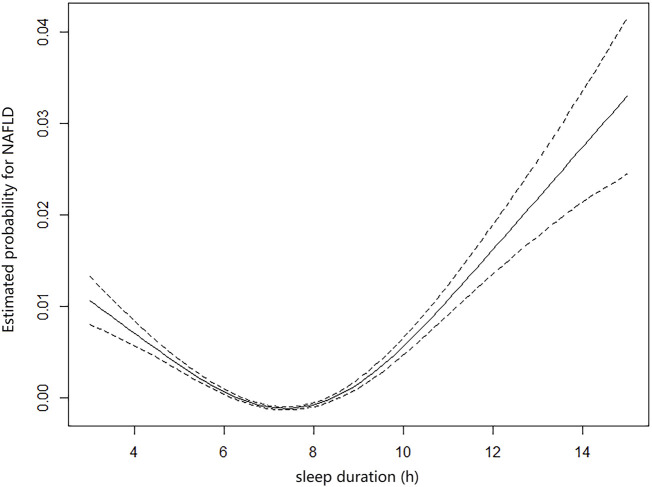
General additive model plotting the U-shaped association between self-reported sleep duration and probabilities of prevalent NAFLD (sleep duration <3 h and >15 h were excluded).

The multivariable regression model adjusted for age and sex showed a significant association between the seven sleep traits and NAFLD (Figure 1; Table 2). After full adjustment, NAFLD was significantly associated with long sleep (HR, 1.22; 95% CI, 1.01–1.48), getting up in the morning not very easy (HR, 1.33; 95% CI, 1.02–1.74) and not at all easy (HR, 1.51; 95% CI, 1.27–1.78), usually nap during day (HR, 1.28; 95% CI, 1.02–1.61), usually insomnia (HR, 1.24; 95% CI, 1.04–1.48), and sometimes insomnia (HR, 1.46; 95% CI, 1.21–1.75) (Table 2). After the FDR was adjusted, NAFLD was significantly associated with getting up in morning not at all easy and usually insomnia (FDR adjusted *p* < 0.007). Furthermore, the easiness of getting up in the morning and insomnia showed a dose–response association with NAFLD (P_trend_ <0.05). The general additive model plotted a U-shaped association between sleep duration and the probability of prevalent NAFLD (Figure 2).

**TABLE 2:** Multivariable Cox proportional hazards model analysis for risk of nonalcoholic fatty liver disease and sleep traits (7 × 3 different models).

Sleep traits	Multivariable model 1		Multivariable model 2		Multivariable model 3	
	HR (95%CI)	*p*	HR (95%CI)	*p*	HR (95%CI)	*p*
Sleep duration						
7–8 h	1.00		1.00		1.00	
<7 h	1.32 (1.15–1.52)	<0.001	1.09 (0.94–1.25)	0.251	1.06 (0.92–1.22)	0.430
>8 h	1.68 (1.38–2.04)	<0.001	1.26 (1.04–1.54)	0.020	1.22 (1.01–1.48)	0.048
P for trend		0.162		0.969		0.778
Easiness of getting up in the morning						
Fairy easy	1.00		1.00		1.00	
Very easy	0.99 (0.86–1.15)	0.929	0.99 (0.86–1.15)	0.956	1.01 (0.88–1.17)	0.859
Not very easy	1.73 (1.47–2.05)	<0.001	1.49 (1.14–1.94)	0.003	1.33 (1.02–1.74)	0.035
Not at all easy	2.17 (1.67–2.82)	<0.001	1.52 (1.29–1.80)	<0.001	1.51 (1.27–1.78)	<0.001
P for trend		<0.001		<0.001		<0.001
Chronotype						
Definitely a ‘morning’ person	1.00		1.00		1.00	
More ‘morning’	0.84 (0.71–0.98)	0.029	0.91 (0.77–1.07)	0.250	0.91 (0.77–1.07)	0.249
More ‘evening’	1.16 (0.99–1.36)	0.074	1.11 (0.89–1.39)	0.359	1.13 (0.97–1.33)	0.125
Definitely an ‘evening’ person	1.24 (0.99–1.56)	0.058	1.16 (0.99–1.37)	0.057	1.06 (0.84–1.33)	0.621
P for trend		0.004		0.038		0.103
Nap during day						
Never/rarely	1.00		1.00		1.00	
Sometimes	1.50 (1.32–1.71)	<0.001	1.18 (1.03–1.34)	0.016	1.11 (0.97–1.26)	0.128
Usually	2.24 (1.79–2.80)	<0.001	1.44 (1.14–1.80)	0.002	1.28 (1.02–1.61)	0.032
P for trend		<0.001		<0.001		0.021
Insomnia						
Never/rarely	1.00		1.00		1.00	
Sometimes	1.38 (1.15–1.64)	<0.001	1.29 (1.08–1.53)	0.005	1.24 (1.04–1.48)	0.018
Usually	2.01 (1.67–2.41)	<0.001	1.53 (1.28–1.84)	<0.001	1.46 (1.21–1.75)	<0.001
P for trend		<0.001		<0.001		<0.001
Snoring						
No	1.00		1.00		1.00	
Yes	1.48 (1.31–1.68)	<0.001	1.13 (0.99–1.28)	0.064	1.07 (0.94–1.21)	0.299
Narcolepsy						
Never/rarely	1.00		1.00		1.00	
Sometimes	1.39 (1.21–1.60)	<0.001	1.14 (0.99,1.32)	0.068	1.10 (0.96–1.27)	0.184
Often	1.90 (1.40–2.58)	<0.001	1.20 (0.88–163)	0.243	1.17 (0.86–1.60)	0.306
P for trend		<0.001		<0.001		0.048

Multivariable model 1 was adjusted for age and sex; multivariable model 2 was adjusted for age, sex, BMI, T2D, and hypertension. Multivariable model 3 was adjusted for age, sex, ALT, AST, waist circumference, hip circumference, BMI, T2D, primary hypertension, HDL, LDL, TG, TC, glucose, and smoking status.

HR, hazard ratio; 95% CI, 95% confidence interval.

### Mendelian Randomization Analysis

After harmonization of the SNP effects, 47 and 9 SNPs were employed as IVs for insomnia and NAFLD, respectively, in the bi-directional MR analysis. The MR-PRESSO outlier test identified no outlier SNPs The *F* statistics of insomnia and NAFLD were 31.802 and 55.156, respectively, indicating that the none of our IVs was subject to weak instrument bias, as each *F* statistic was >10 (Supplementary Table S4) (Burgess and Thompson, 2011; Bull et al., 2020).

In the MR analyses of sleep traits on NAFLD, no genetic evidence of causal effects of insomnia on NAFLD was identified (Table 3). In the MR analyses of NAFLD on sleep traits, NAFLD had a causal effect on easiness of getting up in the morning (IVW; OR, 0.995; 95% CI, 0.990–0.999; *p* = 0.033) and insomnia (IVW; OR, 1.006; 95% CI, 1.001–1.011; *p* = 0.024) (Table 3). Cochran’s Q test and MR-Egger intercept revealed no pleiotropic effect or horizontal heterogeneity that existed in the bi-directional MR analyses (Supplementary Table S4). The results of the leave-one-out analysis suggested that no individual SNP was responsible for driving the observed associations (Supplementary Figures S2–4).

**TABLE 3:** Association between genetically predicted insomnia on nonalcoholic fatty liver and genetically predicted nonalcoholic fatty liver on insomnia and easiness of getting up in the morning used as instrument variables.

Exposure/outcome	Method	SNPs	*p*	OR (95% CI)
Insomnia on NAFLD	MR-Egger	47/57	0.472	0.743 (0.332–1.661)
	Weighted Median		0.746	0.982 (0.879–1.097)
	IVW		0.613	0.976 (0.890–1.071)
	Simple mode		0.095	0.678 (0.433–1.059)
	Weighted mode		0.630	0.976 (0.887–1.075)
NAFLD on insomnia	MR-Egger	9/12	0.207	1.014 (0.995–1.033)
	Weighted Median		0.035	1.007 (1.001–1.014)
	IVW		0.024	1.006 (1.001–1.011)
	Simple mode		0.103	1.009 (0.999–1.018)
	Weighted mode		0.072	1.009 (1.001–1.018)
NAFLD on easiness of getting up in the morning	MR-Egger	9/12	0.879	0.999 (0.981–1.016)
	Weighted Median		0.073	0.995 (0.989–1.0004)
	IVW		0.033	0.995 (0.990–0.999)
	Simple mode		0.126	0.993 (0.986–1.001)
	Weighted mode		0.145	0.994 (0.986–1.001)

In the SNPs column, the number after “/” represents the number of SNPs initially included in the study, and the number before “/” represents the number of SNPs finally employed after harmonization of the SNP effects.

IVW, inverse variance weighted; W median, weighted median; S mode, simple mode; W mode, weighted mode; MR, Mendelian randomization.

## Discussion

This study included seven sleep traits in the UK Biobank, covering almost all sleep phenotypes and comprehensively evaluated the observational associations between seven sleep traits and NAFLD. Meanwhile, we used bidirectional two-sample MR to estimate the bidirectional causal effects between sleep traits (ease of getting up in the morning and insomnia) and NAFLD, providing more reliable causal evidence.

Previous studies suggested that short sleep and long sleep durations increased the risk of NAFLD (Liu et al., 2016; Peng et al., 2017; Kim et al., 2018; Okamura et al., 2019), while sleeping 6–8 h per day lowered the risk of liver steatosis (Mikolasevic et al., 2020). However, the effects were inconsistent across studies (Miyake et al., 2015; Liu et al., 2016; Shen et al., 2016). This may be because previous studies are biased with potential confounding and limited sample size. Our study adopted individual-level data from the UK Biobank with a large sample size and strict data quality control, with findings that 7–8 h of sleep was associated with the lowest risk of NAFLD.

The present study used multivariable regression approach to adjust different covariates, which could provide independent associations. Findings of the observational analyses showed that trouble getting up in the morning and usually insomnia were significantly associated with an increased risk of NAFLD. Moreover, the observational associations were consistent with the causal effects of the MR analyses. The MR analyses highlight the importance of genetically predicted NAFLD on insomnia and easiness of getting up in the morning, which provides a possible target for further mechanism research. People who suffer from insomnia often have trouble getting up in the morning (Danielsson et al., 2019). However, trouble getting up in the morning received much less attention and a lack of drug development than insomnia, which might be an important direction for future research.

Our analysis also identified some clinically significant sleep traits, including narcolepsy, snoring, chronotype, napping during day, and narcolepsy. The regression model adjusted for age and sex showed that evening preference, snoring, long sleep, nap during day, and narcolepsy individuals had a higher risk of NAFLD, and the correlation disappeared after adjusting for the 15 potential confounders. However, given the results of previous studies and the correlation between the sleep traits (Peng et al., 2017; Alshaer et al., 2019; Wang et al., 2020), evening preference, snoring, long sleep, nap during day, and narcolepsy remain clinically important for NAFLD. A previous study showed that a longer daytime napping duration was associated with NAFLD in a dose-dependent manner (Peng et al., 2017). Our findings corroborate that daytime napping is associated with NAFLD risk, but because napping during day increases sleep duration, it is not clear whether daytime naps directly affect NAFLD or affect sleep duration. Unfortunately, the duration of daytime naps was not available in the UK Biobank, precluding further analyses.

Many previous reports have employed MR studies to determine the causal relationship between sleep-related problems and various diseases, such as cardiovascular (Zheng et al., 2019) and several types of cancer (Richmond et al., 2019; Titova et al., 2020). However, the existing evidence on sleep traits and NAFLD are predominantly from cross-sectional studies involving reverse causality (Musso et al., 2013; Liu et al., 2016; Peng et al., 2017; Wijarnpreecha et al., 2017; Kim et al., 2018; Okamura et al., 2019; Wang et al., 2020), and no MR study has assessed the causal relationship between sleep traits and NAFLD, regardless of increasing sleep-related diseases. Our study used a bi-directional MR approach to examine the causal relationships between NAFLD and sleep traits filling the blanks that current evidence of sleep traits and NAFLD involve reverse causation (Okamura et al., 2019).

There are some strengths and limitations to our study. The major strength of this work is the application of bidirectional MR to explore the causation between sleep traits and NAFLD, which could avoid reverse causation and potential false associations caused by unmeasured confounders. Second, all the IVs we used are from the largest published GWAS, and none of our IVs has been subject to weak instrument bias (*F* statistic >10). Meanwhile, we performed multiple methods to examine pleiotropy and heterogeneity to ensure that the IVs met the core assumptions. Third, we implemented a variety of sensitivity analyses to reduce potential bias and winner’s curse (Munafò et al., 2018; Hughes et al., 2019) and applied multiple methods to estimate the causal effect as cross-validation. One limitation is that multivariable regression might be with overfitting problems. But our study with a large sample size could, to some extent, avoid overfitting. Another potential limitation is that the sleep traits used in this study are self-reported. Therefore, participants may have misunderstood the questions. Insomnia symptoms were not necessarily associated with clinical insomnia (Richmond et al., 2019).

In conclusion, our findings demonstrate that trouble getting up in the morning and insomnia are associated with an increased risk of NAFLD. The MR analysis revealed that the associations were causal of NAFLD on easiness of getting up in the morning and insomnia. Our study has significant implications for understanding the causal relationship between sleep traits and NAFLD; more research on their pathways and interactions are highly warranted.

## Data Availability

The original contributions presented in the study are included in the article/Supplementary Material, and further inquiries can be directed to the corresponding authors.
