# Supplementary material for: Investigating the Association Between Seven Sleep Traits and Nonalcoholic Fatty Liver Disease: Observational and Mendelian Randomization Study
Source: Front Genet. 2022 May 17;13:792558. doi: 10.3389/fgene.2022.792558 (PMC9152285; doi:10.3389/fgene.2022.792558)
Supplement: Supplementary file 1 [file DataSheet1.PDF]

Investigating the Association between seven sleep traits and nonalcoholic fatty liver disease: observational and Mendelian randomization study

Hong Fan[1,2], Zhenqiu Liu[1,2,3], Xin Zhang[1,2], Huangbo Yuan[2], Xiaolan Zhao[4], Renjia Zhao[1,2], Tingting Shi[1,2], Sheng Wu[1,2], Yiyun Xu[1,2], Chen Suo[1,2], Xingdong Chen[2,3,4†], Tiejun Zhang[1, 2†]

1. Department of Epidemiology, School of Public Health, Fudan University, Shanghai, China. Key Laboratory of Public Health Safety (Fudan University), Ministry of Education, China
2. Fudan University Taizhou Institute of Health Sciences, Taizhou, China.
3. State Key Laboratory of Genetic Engineering and Collaborative Innovation Center for Genetics and Development, School of Life Sciences, Fudan University, Shanghai 200438, China.
4. Department of chronic diseases prevention, Taizhou center for disease control and prevention. Taizhou, Jiangsu, China.
5. Human Phenome Institute, Fudan University, 825 Zhangheng Road, Shanghai, China.

†: Correspondence to Tiejun Zhang MD., PhD., Professor of Epidemiology, School of Public Health, Fudan University, Shanghai, 200032, China. Email: tjzhang@shmu.edu.cn; TEL/FAX: +86−21−54237088; or Xingdong Chen MD., PhD.,

School of Life Sciences, Fudan University, Shanghai, 200032, China. Email: xingdongchen@fudan.edu.cn; TEL/FAX: +86-21-51630602.

TABLE OF CONTENTS

SUPPLEMENTARY METHODS…………………………….……..……...3
Inclusion and exclusion criteria for participants……………………………3
Genotyping and imputation ………………………………………..….3

SUPPLEMENTARY FIGURES
Figure S1…………………………………………………………….....4
Figure S2…………………………………………………………….....5
Figure S3…………………………………………………………….....6
Figure S4………………………………………………………………6

SUPPLEMENTARY TABLES
Table S1……………………………………………………………......7
Table S2……………………………………………………………......7
Table S3………………………………………………………………9
Table S4………………………………………………………………10

REFERENCES……..………………………………………………………11

SUPPLEMENTARY METHODS

inclusion and exclusion criteria for participants

In the observational association analysis, we excluded those individuals with a sleep duration less than 3 hours and more than 15 hours that might be outliers, and stratified the remaining sleep duration into short (< 7 hours), intermediate (7–8 hours), and long (>8 hours) groups as 7-8h is the recommended sleep duration [1; 2]. At the same time, the answers which were "Do not know" or "Prefer not to answer" on these seven sleep-related questions were also excluded in the following analysis. In the MR analysis, short sleep was defined as sleep time less than 6 hours and long sleep was defined as sleep time more than 9 hours followed the published genome wide association studies (GWAS)[3].

Genotyping and imputation

Genotyping, primary genotype quality control and imputation were performed by the UK Biobank, which genotyped the samples using BilEVE array or the UK Biobank array[4]. The genotype data were imputed using the HRC reference panel[5] or a merged reference panel consisting of UK10K[6]and 1000 Genomes phase 3[7]

SUPPLEMENTARY TABLE FIGURE

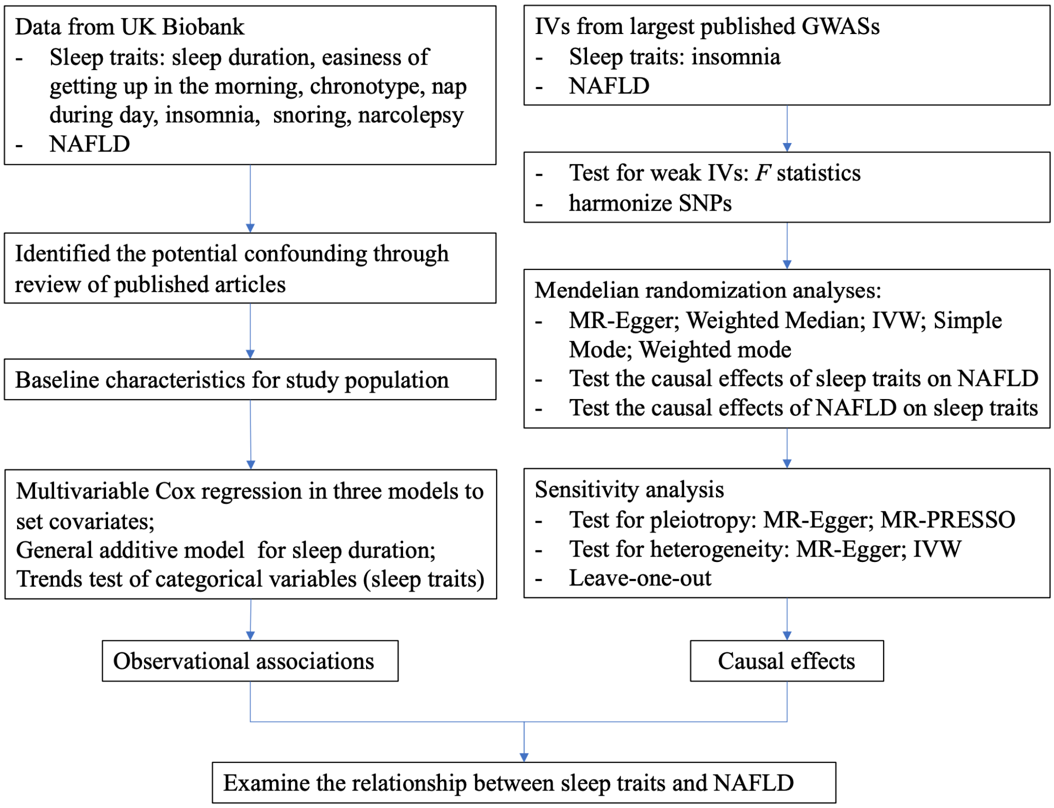

Figure S1. Study design flowchart.

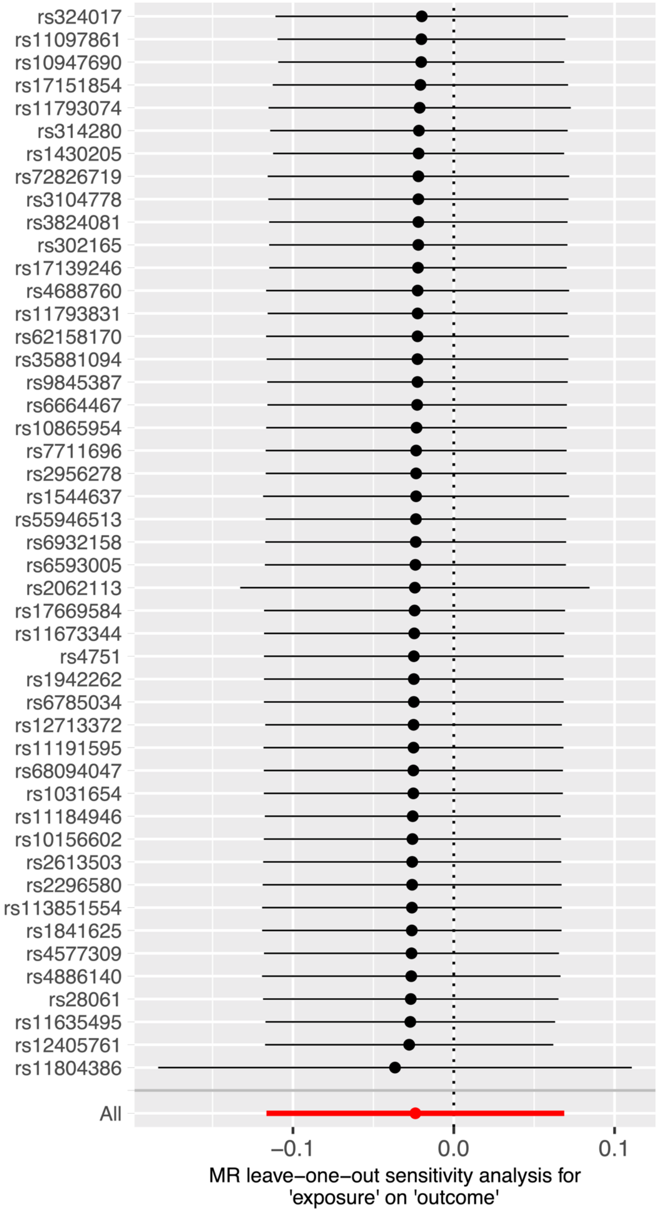

Figure S2. Leave-one-out analysis related to the insomnia genetic instrument with nonalcoholic fatty liver disease.

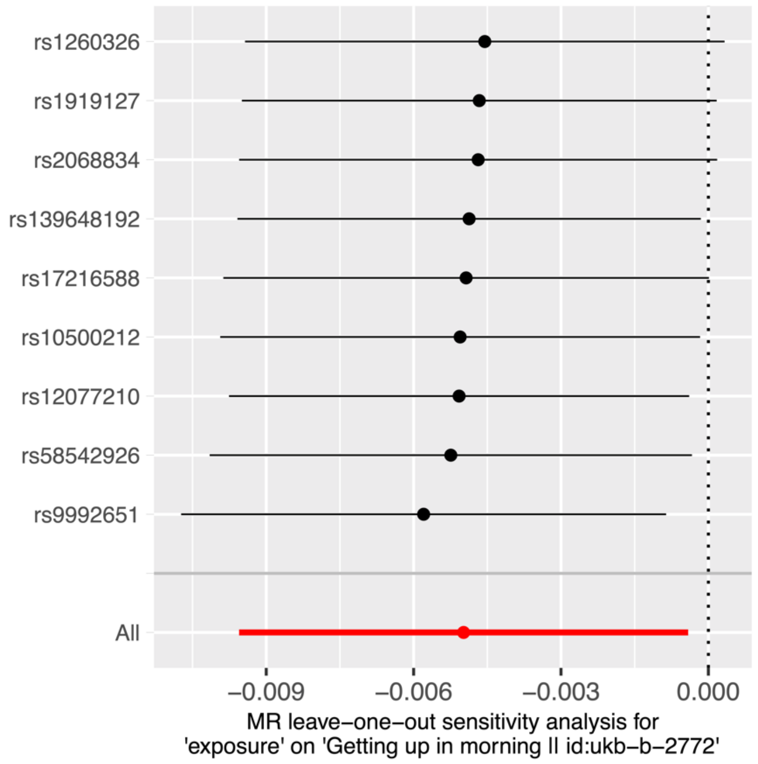

Figure S3. Leave-one-out analysis related to the nonalcoholic fatty liver disease instrument with easiness of getting up in the morning.

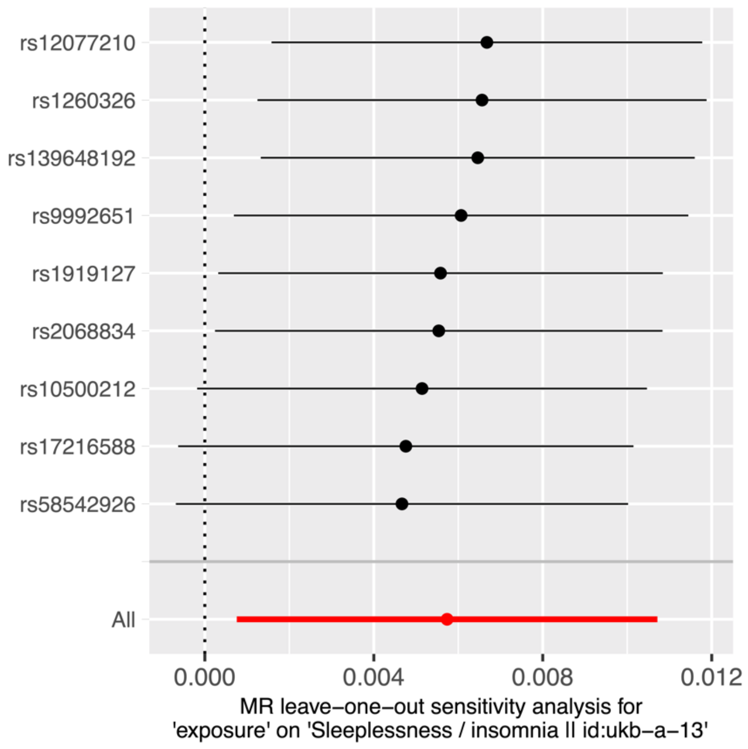

Figure S4. Leave-one-out analysis related to the nonalcoholic fatty liver disease instrument with insomnia.

SUPPLEMENTARY TABLE

Table S1. Characteristics of the GWAS data for instrumental variables.

Sleep traits	nSNP	Ethnic background	Author	year
Insomnia[8]	57	European	Lane JM	2019
NAFLD[9]	12	European	Anstee QM	2020

Table S2. Genetic instrumental variables implemented in the study and their association with each insomnia.

Trait	SNP	Chr:BP	Eff/Alt	EAF	INFO	OR [95% CI]	P Value	Nearest Gene
Insomnia	rs11184946	1:107185225	T/C	0.417	1	1.03 [1.02-1.05]	2.90x10-10	LOC126987/PRMT6
Insomnia	rs6664467	1:151738403	G/A	0.863	0.99	1.05 [1.03-1.06]	4.50x10-8	OAZ3
Insomnia	rs4751	1:1686040	T/G	0.425	1	1.03 [1.02-1.05]	1.60x10-8	NADK
Insomnia	rs2644128	1:201793440	G/C	0.55	1	1.04 [1.03-1.06]	1.00x10-12	NAV1
Insomnia	rs12405761	1:57850914	A/C	0.571	1	1.04 [1.03-1.05]	2.60x10-11	DAB1
Insomnia	rs2613503	1:72839774	A/C	0.802	0.99	1.04 [1.02-1.06]	2.80x10-7	LOC100132353/KRT8P21
Insomnia	rs11804386	1:87738947	A/G	0.332	1	1.04 [1.03-1.06]	2.60x10-8	LOC339524/LMO4
Insomnia	rs62158170	2:114082175	A/G	0.784	0.99	1.05 [1.04-1.08]	5.70x10-13	PAX8/LOC100130100
Insomnia	rs4577309	2:191288833	A/G	0.468	0.99	1.03 [1.02-1.05]	3.70x10-9	FLJ20160
Insomnia	rs12713372	2:58377014	C/T	0.566	0.99	1.02 [1.01-1.04]	1.20x10-5	VRK2
Insomnia	rs35881094	2:58922921	G/T	0.426	0.99	1.05 [1.03-1.06]	3.00x10-15	LOC644456/LOC730134
Insomnia	rs2192338	2:59833168	C/G	0.78	0.99	1.05 [1.03-1.06]	1.20x10-7	LOC647038/BCL11A
Insomnia	rs113851554	2:66750564	T/G	0.058	0.93	1.20 [1.16-1.24]	1.30x10-41	MEIS1
Insomnia	rs72826719	2:66976854	A/G	0.049	0.97	1.10 [1.07-1.14]	2.20x10-12	LINC01797/LINC01799
Insomnia	rs9845387	3:116425935	C/A	0.959	0.99	1.10 [1.06-1.14]	2.10x10-8	BZW1L1/LOC285194
Insomnia	rs6785034	3:184781830	A/G	0.422	1	1.03 [1.02-1.04]	9.60x10-5	VPS8/C3orf70
Insomnia	rs4683301	3:46931478	T/A	0.6	0.99	1.04 [1.02-1.05]	4.60x10-8	PTHR1
Insomnia	rs10865954	3:49211989	T/C	0.334	1	1.04 [1.02-1.05]	1.70x10-9	KLHDC8B
Insomnia	rs4688760	3:49980596	T/C	0.69	0.99	1.05 [1.03-1.06]	1.00x10-12	RBM6
Insomnia	rs55946513	3:52599792	C/T	0.934	1	1.05 [1.02-1.08]	7.30x10-6	PBRM1
Insomnia	rs11097861	4:105330133	G/A	0.715	0.99	1.04 [1.03-1.05]	1.70x10-9	TACR3/CXXC4
Insomnia	rs1841625	4:91287477	G/A	0.432	0.99	1.03 [1.01-1.04]	3.60x10-8	MGC48628
Insomnia	rs28061	5:102543878	A/G	0.692	0.99	1.04 [1.02-1.05]	2.10x10-8	HISPPD1/C5orf30
Insomnia	rs1592757	5:103889998	C/G	0.357	1	1.04 [1.03-1.06]	4.60x10-10	NUDT12/RAB9P1
Insomnia	rs7711696	5:135486536	T/G	0.305	1	1.05 [1.03-1.06]	9.90x10-12	SMAD5
Insomnia	rs1430205	5:87678585	T/C	0.458	1	1.03 [1.02-1.05]	3.60x10-8	LOC100130786/LOC645323

Insomnia	rs6932158	6:101246010	C/T	0.491	1	1.03 [1.02-1.05]	2.80x10-8	ASCC3
Insomnia	rs314280	6:105400837	G/A	0.548	1	1.04 [1.02-1.05]	3.50x10-10	RP3-439I14.1
Insomnia	rs10947690	6:37631768	G/A	0.261	1	1.04 [1.02-1.06]	3.50x10-8	MDGA1
Insomnia	rs3824081	7:1024581	T/C	0.475	0.99	1.04 [1.03-1.06]	1.10x10-8	CYP2W1
Insomnia	rs10280045	7:114076394	G/C	0.574	0.99	1.04 [1.03-1.05]	1.00x10-10	FOXP2
Insomnia	rs302165	7:18323899	G/A	0.216	0.99	1.04 [1.02-1.06]	2.40x10-6	PRPS1L1/HDAC9
Insomnia	rs6593005	7:52584625	G/A	0.741	1	1.03 [1.02-1.05]	8.60x10-9	LOC100131871/LOC100133258
Insomnia	rs17151854	8:10236559	T/G	0.154	0.99	1.06 [1.04-1.08]	2.40x10-8	MSRA
Insomnia	rs11793831	9:23362311	G/T	0.583	1	1.04 [1.02-1.05]	1.50x10-6	FLJ35282/LOC402360
Insomnia	rs11793074	9:23820070	A/G	0.853	1	1.04 [1.02-1.06]	2.30x10-7	ELAVL2
Insomnia	rs10156602	9:96345328	A/G	0.638	0.98	1.04 [1.03-1.05]	3.40x10-12	PHF2
Insomnia	rs2296580	10:104241683	G/T	0.702	1	1.05 [1.04-1.08]	8.70x10-12	ACTR1A
Insomnia	rs11191595	10:104943048	A/C	0.937	0.9	1.10 [1.06-1.14]	1.50x10-9	NT5C2
Insomnia	rs10838708	11:47441513	G/A	0.541	0.98	1.03 [1.02-1.04]	2.50x10-9	PSMC3
Insomnia	rs68094047	12:109855201	T/C	0.25	0.99	1.04 [1.03-1.06]	3.30x10-9	MYO1H
Insomnia	rs324017	12:57487814	A/C	0.295	1	1.05 [1.04-1.07]	1.10x10-10	NAB2/STAT6
Insomnia	rs2956278	12:84698234	G/A	0.215	1	1.05 [1.03-1.07]	1.30x10-8	LOC401725/LOC100128335
Insomnia	rs2147141	13:112707954	G/C	0.543	0.98	1.04 [1.02-1.05]	8.20x10-8	C13orf16/SOX1
Insomnia	rs1923770	13:53786568	T/A	0.383	1	1.05 [1.03-1.06]	2.30x10-12	OLFM4/RP11-365K22.1
Insomnia	rs1031654	13:54382035	C/A	0.202	1	1.05 [1.03-1.07]	2.00x10-9	LOC100133285/RP11-365K22.1
Insomnia	rs4886140	13:59833519	G/A	0.668	0.98	1.04 [1.03-1.06]	3.10x10-10	LOC100129308/DIAPH3
Insomnia	rs11635495	15:67804682	C/T	0.515	1	1.04 [1.02-1.05]	6.80x10-9	IQCH/C15orf61
Insomnia	rs4886860	15:74340336	G/C	0.234	1	1.05 [1.03-1.06]	6.10x10-12	PML
Insomnia	rs1544637	16:51484837	T/C	0.488	0.99	1.03 [1.02-1.04]	3.00x10-8	UNGP1/LOC642659
Insomnia	rs3104778	16:52633652	A/G	0.589	0.99	1.03 [1.01-1.04]	4.20x10-8	LOC643714
Insomnia	rs2062113	16:59476179	T/C	0.43	1	1.04 [1.02-1.05]	1.90x10-10	LOC644600/LOC644649
Insomnia	rs17139246	16:6106260	C/T	0.389	0.98	1.03 [1.02-1.05]	4.10x10-8	RBFOX1
Insomnia	rs17669584	17:28899614	G/A	0.195	0.96	1.04 [1.03-1.06]	3.60x10-8	LOC729704
Insomnia	rs11651809	17:43255681	G/C	0.296	0.98	1.05 [1.04-1.07]	2.10x10-15	HEXIM2/LOC339192
Insomnia	rs1942262	18:52873317	A/G	0.292	0.99	1.04 [1.03-1.06]	1.10x10-13	CCDC68/TCF4
Insomnia	rs11673344	19:37684966	G/A	0.38	1	1.04 [1.03-1.06]	9.00x10-10	ZNF585B

Table S3. Genetic instrumental variables implemented in the study and their association with each nonalcoholic fatty liver disease.

Trait	SNP	chr	Effect allele	Other allele	gene	pval	OR	CI
NAFLD	rs12077210	1	T	C	LEPR	5.62E-08	1.484	(1.287, 1.711)
NAFLD	rs1260326	2	T	C	GCKR	1.06E-10	1.278	(1.186, 1.377)
NAFLD	rs1919127	2	C	T	C2orf16	5.61E-10	1.29	(1.190, 1.398)
NAFLD	rs2068834	2	C	T	ZNF512	8.49E-11	1.302	(1.202, 1.410)
NAFLD	rs9992651	4	A	G	HSD17B13	2.78E-08	0.744	(0.671, 0.826)
NAFLD	rs13118664	4	T	A	HSD17B13	1.41E-08	0.74	(0.667, 0.821)
NAFLD	rs139648192	8	T	C	-	5.20E-08	1.538	(1.317, 1.796)
NAFLD	rs58542926	19	T	C	TM6SF2	2.05E-11	1.609	(1.400, 1.849)
NAFLD	rs8107974	19	T	A	SUGP1	2.58E-12	1.632	(1.423, 1.872)
NAFLD	rs17216588	19	T	C	-	7.25E-14	1.612	(1.423, 1.827)
NAFLD	rs10500212	19	T	C	PBX4	3.40E-12	1.549	(1.369, 1.752)
NAFLD	rs738409	22	G	C	PNPLA3	1.45E-49	1.827	(1.687, 1.979)

Table S4. Heterogeneity test and pleiotropy test for instruments

Sleep trait	F statistic	MR-PRESSO		Cochran's Q test (MR Egger)		Cochran's Q test (IVW)		Horizontal pleiotropy tests		
		RSSobs	P presso	Q	P value	Q	P Value	Intercept	se	P value
**Sleep traits on NAFLD**										
Insomnia	31.802	64.110	0.282	51.517	0.234	52.032	0.251	0.011	0.016	0.506
**NAFLD on sleep traits**										
Easiness of getting up in morning	55.156	5.956	0.939	1.056	0.994	1.231	0.996	-0.001	0.003	0.688
Insomnia		19.607	0.189	6.090	0.529	6.772	0.561	-0.003	0.003	0.436

References

[1] M. Hirshkowitz, K. Whiton, S.M. Albert, C. Alessi, O. Bruni, L. DonCarlos, N. Hazen, J. Herman, P.J. Adams Hillard, E.S. Katz, L. Kheirandish-Gozal, D.N. Neubauer, A.E. O'Donnell, M. Ohayon, J. Peever, R. Rawding, R.C. Sachdeva, B. Setters, M.V. Vitiello, and J.C. Ware, National Sleep Foundation's updated sleep duration recommendations: final report. Sleep health 1 (2015) 233-243.

[2] X. Li, Q. Xue, M. Wang, T. Zhou, H. Ma, Y. Heianza, and L. Qi, Adherence to a Healthy Sleep Pattern and Incident Heart Failure: A Prospective Study of 408802 UK Biobank Participants. Circulation (2020).

[3] H.S. Dashti, and S.E. Jones, Genome-wide association study identifies genetic loci for self-reported habitual sleep duration supported by accelerometer-derived estimates. Nature communications 10 (2019) 1100.

[4] C. Bycroft, C. Freeman, D. Petkova, G. Band, L.T. Elliott, K. Sharp, A. Motyer, D. Vukcevic, O. Delaneau, J. O'Connell, A. Cortes, S. Welsh, A. Young, M. Effingham, G. McVean, S. Leslie, N. Allen, P. Donnelly, and J. Marchini, The UK Biobank resource with deep phenotyping and genomic data. Nature 562 (2018) 203-209.

[5] S. McCarthy, and S. Das, A reference panel of 64,976 haplotypes for genotype imputation. Nature genetics 48 (2016) 1279-83.

[6] K. Walter, J.L. Min, J. Huang, L. Crooks, Y. Memari, S. McCarthy, J.R. Perry, C. Xu, M. Futema, D. Lawson, V. Iotchkova, S. Schiffels, A.E. Hendricks, P. Danecek, R. Li, J. Floyd, L.V. Wain, I. Barroso, S.E. Humphries, M.E. Hurles, E. Zeggini, J.C. Barrett, V. Plagnol, J.B. Richards, C.M. Greenwood, N.J. Timpson, R. Durbin, and N. Soranzo, The UK10K project identifies rare variants in health and disease. Nature 526 (2015) 82-90.

[7] G.R. Abecasis, D. Altshuler, A. Auton, L.D. Brooks, R.M. Durbin, R.A. Gibbs, M.E. Hurles, and G.A. McVean, A map of human genome variation from population-scale sequencing. Nature 467 (2010) 1061-73.

[8] J.M. Lane, and S.E. Jones, Biological and clinical insights from genetics of insomnia symptoms. Nature genetics 51 (2019) 387-393.

[9] Q.M. Anstee, R. Darlay, S. Cockell, M. Meroni, O. Govaere, D. Tiniakos, A.D. Burt, P. Bedossa, J. Palmer, Y.L. Liu, G.P. Aithal, M. Allison, H. Yki-Järvinen, M. Vacca, J.F. Dufour, P. Invernizzi, D. Prati, M. Ekstedt, S. Kechagias, S. Francque, S. Petta, E. Bugianesi, K. Clement, V. Ratziu, J.M. Schattenberg, L. Valenti, C.P. Day, H.J. Cordell, and A.K. Daly, Genome-wide association study of non-alcoholic fatty liver and steatohepatitis in a histologically characterised cohort(☆). J Hepatol 73 (2020) 505-515.
